# Computer-Assisted Planning and Patient-Specific Instruments for Bone Tumor Resection within the Pelvis: A Series of 11 Patients

**DOI:** 10.1155/2014/842709

**Published:** 2014-07-02

**Authors:** François Gouin, Laurent Paul, Guillaume Anthony Odri, Olivier Cartiaux

**Affiliations:** ^1^Clinique Chirurgicale Orthopédique et Traumatologique, CHU Hôtel-Dieu, Place Alexis-Ricordeau 1, 44093 Nantes Cedex 1, France; ^2^Laboratoire Physiopathologie de la Résorption Osseuse, Inserm UI957, Faculté de Medecine, Université de Nantes, rue Gaston Veil, 44000 Nantes, France; ^3^Computer Assisted and Robotic Surgery (CARS), Institut de Recherche Expérimentale et Clinique, Université Catholique de Louvain, Avenue Mounier 53, 1200 Brussels, Belgium

## Abstract

Pelvic bone tumor resection is challenging due to complex geometry, limited visibility, and restricted workspace. Accurate resection including a safe margin is required to decrease the risk of local recurrence. This clinical study reports 11 cases of pelvic bone tumor resected by using patient-specific instruments. Magnetic resonance imaging was used to delineate the tumor and computerized tomography to localize it in 3D. Resection planning consisted in desired cutting planes around the tumor including a safe margin. The instruments were designed to fit into unique position on the bony structure and to indicate the desired resection planes. Intraoperatively, instruments were positioned freehand by the surgeon and bone cutting was performed with an oscillating saw. Histopathological analysis of resected specimens showed tumor-free bone resection margins for all cases. Available postoperative computed tomography was registered to preoperative computed tomography to measure location accuracy (minimal distance between an achieved and desired cut planes) and errors on safe margin (minimal distance between the achieved cut planes and the tumor boundary). The location accuracy averaged 2.5 mm. Errors in safe margin averaged −0.8 mm. Instruments described in this study may improve bone tumor surgery within the pelvis by providing good cutting accuracy and clinically acceptable margins.

## 1. Introduction

Limb salvage surgery is now the preferred procedure for most patients with bone tumors of pelvis and the lower limb. However, resection of bone tumors within the pelvis remains highly challenging because of the complex three-dimensional (3D) geometry of the pelvic bone and the proximity of important organs and neurovascular structures. This complex and restricted working space can explain the high complication rate usually observed in pelvic bone tumor surgery, particularly the local recurrence rate ranging from 28 to 35% [1]. Accurate resections with wide margins are required since it is highly associated with a low local recurrence rate [2–7].

A previous study investigated the ability of experienced surgeons to perform wide margins during simulated tumor cutting of the pelvis [8]. This in vitro study, while performed under optimal conditions, clearly demonstrated that freehand cutting of bone tumors of the pelvis is not accurate enough to ensure wide margins: the errors on the desired safe margin averaged 5.3 mm with a standard deviation ranging from 2.7 to 5.3 mm among the experienced surgeons, and two resections (out of twelve) were intralesional.

Intraoperative navigation systems have been developed for bone tumor surgery, specifically within the pelvis [9–12]. Several authors have reported their experience in using navigation technology to resect bone tumors [13–21]. Their results are highly encouraging since significant improvements in surgical margins can be observed. A significantly decreased local recurrence rate has been shown by Jeys et al. [18] but should be confirmed with a long-term follow-up (13 months in their clinical series). The value-added of the navigation technology has been recently investigated in an experimental study that demonstrated a significantly improved cutting accuracy during simulated bone tumor surgery of the pelvis [9].

Patient-specific instruments (PSI) have been developed as an alternative to navigation systems. PSI were developed originally for total knee arthroplasty [22, 23] with some residual controversy in terms of the achieved bone-cutting accuracy [24, 25]. Recently, other PSI-assisted applications such as pedicle screw insertion [26–28], hip arthroplasty [29, 30], and corrective osteotomy [31–33] have been described in the literature. PSI technology has been adapted also for bone tumor surgery [34–36]: the patient-specific cutting guides are equipped with bone-specific surfaces to fit into unique position on the patient and flat surfaces to materialize the desired resection planes. A recent experimental study has assessed quantitatively an equivalent value-added of both PSI and navigation technologies in terms of the achieved surgical margins during simulated bone tumor resections of the pelvis [37].

The present study aims to report a series of 11 clinical cases of PSI-assisted bone tumor surgery within the pelvis, with the specific goal of assessing how accurately a preoperative resection strategy can be replicated intraoperatively.

## 2. Materials and Methods

### 2.1. Patient Series

The prospective series was composed of 11 patients eligible for curative surgical resection of primary bone tumor of the pelvis (Table 1). Eight patients had a bone sarcoma of iliac bone involving the acetabulum, two patients had a sacral tumor, and one patient had a chondrosarcoma of proximal femur with intra-articular extension. During the same period, two more patients have been operated on by the same surgeon without PSI, the first because the short delay between the acquisition of CT and MRI images and the surgery did not allow to perform the preoperative planning and the PSI manufacturing and the second because the tumor was a chondrosarcoma arising around a total hip prosthesis that rendered the MRI images useless for the planning process.

### 2.2. Preoperative Planning of the Tumor Resections

The planning of the resection strategy, as described in [13], is based on the MRI and CT images. If these modalities have not been acquired during the diagnosis, a specific exam should be prescribed. Any scanning sequence of the acquired MRI can be used to perform the planning. The CT being crucial for PSI accuracy, its spatial resolution must be 1 mm or below in the *Z* direction. Tumor delineation is made by the surgeon with the aid of a radiologist on the 2D MRI slices using a segmentation software (ITK-Snap, version 2.0.0, Philadelphia) [38]. The set of MRI images was reconstructed in 3D and brought into the coordinate system of the CT images using a homemade multimodality registration algorithm. Then, the 3D model of the tumor volume was registered with the 3D bony structure extracted from the CT images.

**Figure 1:**
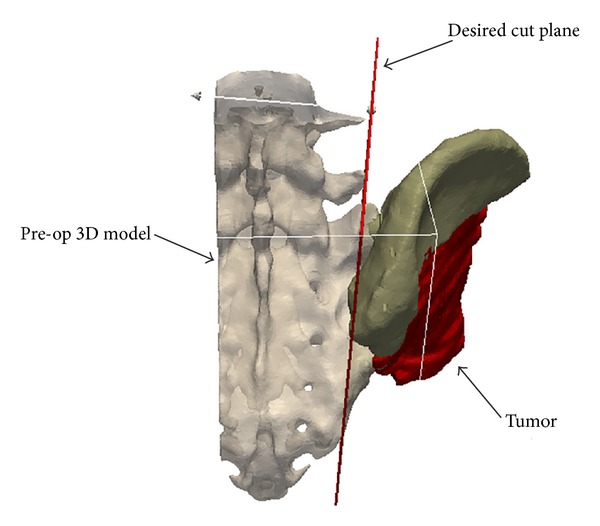
Preoperative planning for patient number 2. Preoperative CT images of the patient were segmented to construct the 3D virtual models of the patient and the tumor. The resection strategy consisted of one target plane defining the desired resection plane with a 6 mm safe margin.

The 3D model of the bony structure with the registered tumor volume was loaded into a visualization and computing software (Paraview, version 3.14.1, New York). This software enabled the surgeon to position target planes close to the boundary of the tumor (from 1 up to 6 planes; Figure 1). For each patient, the resection strategy consisted in several target planes defining the desired bone-cutting with a safe margin defined by the surgeon (namely, 10 to 15 mm). The safe margins have been decreased to 3 mm according to the possibility of preserving anatomic structures and the effectiveness of adjuvant therapy. For instance, for patient number 9, an unusual 3 mm safe margin was defined in order to preserve the sacroiliac joint.

Moreover, sometimes, no safe margin can be easily defined. For example, for patient number 6, the tumor was located in the proximal femur with intra-articular extension, and the resection planning consisted in a 3-plane bone-cutting around the acetabulum, free of tumor, to achieve an extra-articular resection of the hip. Consequently, no desired safe margin has been specifically defined for this case. There was no incidence on the postoperative analysis since the patient was not included because of artifacts created by the metallic implant.

### 2.3. Patient-Specific Instruments

**Figure 2:**
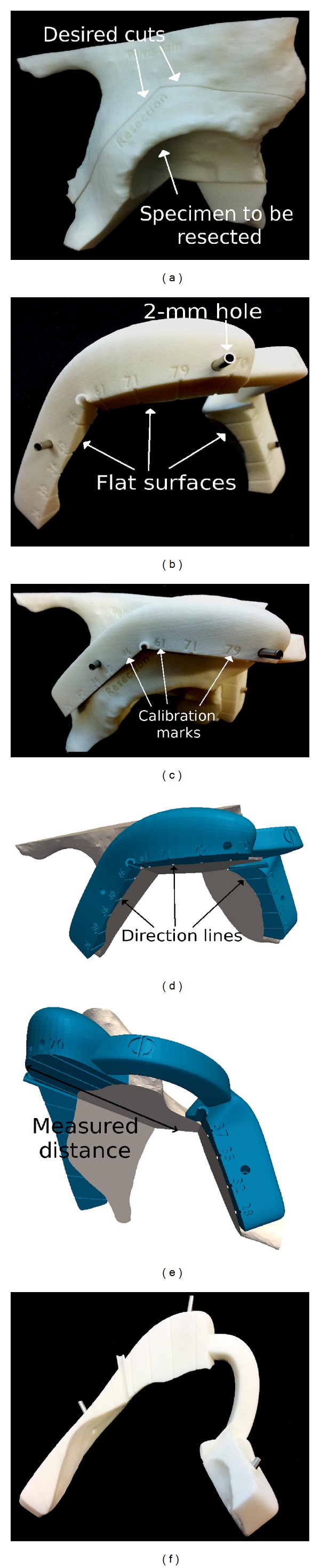
Bone models and PSI produced by additive manufacturing for patient number 6. (a) Bone model of the patient enables the visualization of the desired resection strategy and the tumor specimen to be resected. (b) PSI is equipped with flat surfaces to indicate the desired resection planes, holes to be pinned temporarily on the bone using Kirschner wires. (c) PSI has a position of best fit on the bone model. Calibration marks are engraved on the edge to provide visual control of the cutting depth. (d) Associated with a calibration mark direction lines indicate the depth of cutting. (e) The depth is measured from the outer edge of PSI to the deepest bone structure. (f) The direction lines engraved onto the flat surfaces of PSI.

Patient-specific instruments (PSI) were designed using computer-aided design (CAD) software (Blender version 2.65) according to the desired resection strategy. PSI were designed to have bone-specific contact surfaces to fit into unique position on the bony structure of the patient. These contact surfaces were defined by both surgeon and engineer accounting for surgical approach and bone exposure (described below). PSI were equipped with flat surfaces to indicate the target planes and cylindrical guides for 2 mm diameter Kirschner wires to be pinned on the bony structure (Figure 2(b)). In addition, PSI provided calibration marks that provided a control over the cutting depth to prevent a soft tissue tear by the saw. The marks represented the distance along the associated direction line measured between the outer edge of the PSI and the deepest bone structure to be cut. The intended meaning is illustrated in Figure 2(e). PSI and bone models were manufactured by additive manufacturing with a dimensional tolerance of 0.2 mm using an ISO-certified biocompatible polyamide material [39]. PSI and bone models were sterilized using standard autoclave as recommended by polyamide provider.

### 2.4. PSI-Assisted Tumor Resections

The standard surgical approach has been used for each patient. Soft tissues were dissected following the surgeon's routine technique. Bone was exposed in the area of cuttings before actually performing the resection. The exact dissection areas were identified by using the bone models. PSI required a limited extra bone exposure (less than 10 mm) as their thickness did not exceed 20 mm specifically when positioned in critical area such as under the gluteus medius muscle for an iliac wing section.

**Figure 3:**
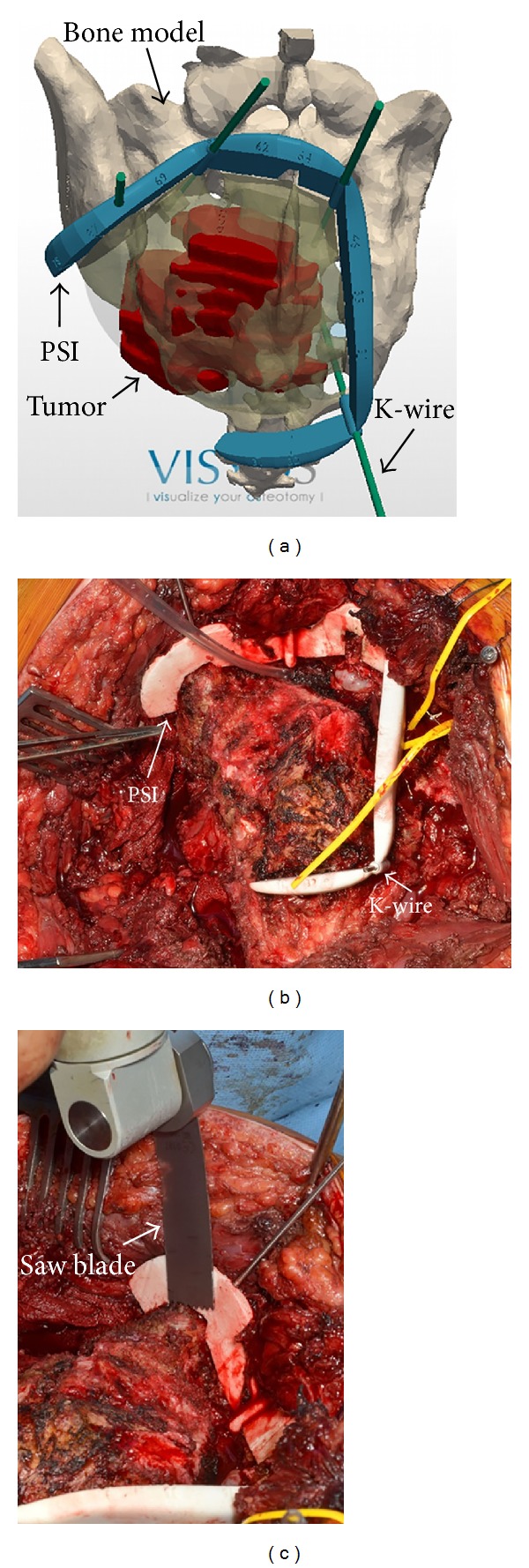
Intraoperative situation for patient number 11. (a) PSI is designed using computer-aided-design software. (b) PSI are sterilizable to be manipulated by the surgeon in the operating room. PSI is positioned on the bone and temporarily fixed using Kirschner wires. (c) Cuts are initiated with the oscillating saw guided by the flat surfaces of the PSI.

PSI was positioned freehand by the surgeon and fixed on the bone surface using the K-wires (Figure 3). Positioning of PSI has been rated intraoperatively by the surgeon using a 4-level scale: excellent (correct position at the first trial, in few seconds, without any doubt), good (easily positioned but not at the first trial), difficult (several trials with peroperative checks on the bone model to achieve a stable positioning), or ambiguous (impossible to find the unique positioning). The flat surfaces materializing the desired resection planes served as mechanical support for the cutting tools, such as an oscillating saw to initiate the bone cuts and then a bone chisel to complete the bone cuts. When the resection was achieved, both K-wires and PSI were taken off, the tumor was mobilized, and intrapelvic tissues were dissected for final* en bloc* extraction of the tumor.

### 2.5. Histopathological Analysis of Resected Specimens

Histopathological analysis of the resected tumor specimens was performed to evaluate the safety of the achieved surgical margins using the standardized classification by the Union for International Cancer Control (UICC). The UICC classification distinguishes R0 as* in sano* resection (SM > 1 mm), R1 as possible microscopic residuals (SM between 0 and 1 mm), and R2 as macroscopic residual disease [40].

### 2.6. Postoperative Follow-Up and Clinical Outcomes

Patients were clinically reviewed every 4 to 6 months. Patients underwent postoperative MRI and CT to assess local control and lung X-ray to control a potential distant spreading of the disease.

### 2.7. Quantitative Evaluation of Bone-Cutting Accuracy

Two parameters were used to evaluate the bone-cutting accuracy. First, the achieved surgical margin (SM) was used to evaluate the accuracy of the bone cut relative to the bone tumor. SM was defined as the minimum distance (mm) between the achieved cut plane and the boundary of the tumor. Consequently, the error in the desired safe margin (ESM) was defined as the difference (mm) between SM and the desired safe margin. Thus, negative values of ESM were found for cutting under the desired safe margin and positive values were found for cutting over the desired safe margin.

Second, the location accuracy (L) was used in accordance with the ISO1101 standard [41, 42] to evaluate the geometrical accuracy of the achieved cut plane with respect to the desired resection plane (the target plane). L was defined as the maximum distance (mm) between the cut plane and the target plane. The evaluation parameters SM and L have been already validated for use in bone tumor surgery in a previous experimental study [37].

**Figure 4:**
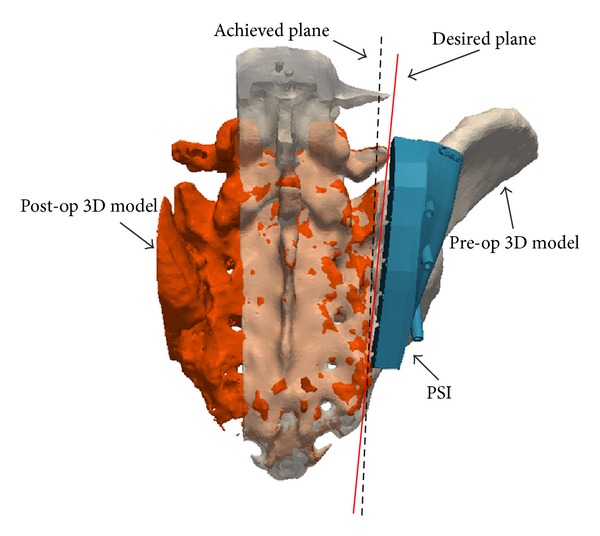
Quantitative evaluation of bone cuts for patient number 2. Postoperative 3D virtual model of the patient was constructed from the postoperative CT images and registered to the preoperative 3D model. The achieved cut plane was manually identified and compared to the desired cut plane. See text for details on the computation of location accuracy parameter L and surgical margin SM.

CT-scans of the patients have been acquired postoperatively. For each CT-scan, the bone surfaces were extracted using ITK-Snap segmentation software. Then, each postoperative 3D model of the patient was loaded in Paraview visualization software and registered manually with the corresponding preoperative 3D model and planning (Figure 4). Accurate registration between pre- and postoperative 3D models was validated through visual inspection of the data.

One operator measured the parameters SM and L using Paraview visualization software. The operator measured SM by defining the closest point of each cut plane from the boundary of the tumor. Then the operator measured L by defining the most distant point of each cut plane from the corresponding target plane and measuring numerically the distance along the normal of the target plane. For patient number 2, the parameter L had to be corrected by the thickness of the saw blade to account for the loss of bone material (the kerf) during bone-cutting [43], because the PSI was positioned on the tumor side.

Results are presented as the mean and 95% confidence interval (CI).

## 3. Results

### 3.1. Histopathological Results and Clinical Outcomes

Positioning of the PSI on the bone surface was unambiguous for all cases. The positioning has been rated as excellent in seven patients, good in three patients, and difficult in one patient. Finally, the PSI was positioned within 5 minutes for each case.

All achieved surgical margins were classified R0, except in two patients. Patient number 5 suffered from a tumor in close contact with external iliac vessels. Although bone margins were classified R0, the resection was classified R1 because soft tissues margin was considered between 0 and 1 mm. Patient number 8 suffered intraoperatively from bad cardiovascular condition associated with severe bleeding requiring urgent extraction of the tumor which has been consequently morselized. The surgical margin was then classified R2.

The postoperative follow-up averaged 14 months with a range from 0 to 28 months. At the time of follow-up, patient number 5 had a recurrence at 18 months around iliac vessels. Reoperation was performed with soft tissue resection including vessels with allograft arterial reconstruction.

### 3.2. Bone-Cutting Accuracy

Of the 26 cut planes performed by the surgeon in this study, nine cut planes were eligible for the evaluation of the bone-cutting accuracy (Table 2). Twelve cut planes (all the cut planes of patients numbers 3, 6, and 8 and two cut planes of patient number 11) were excluded from the evaluation process because of no available postoperative CT-scan or inadequate resolution. One cut plane (patient number 1) was excluded because the presence of the bone autograft (reconstruction with hip transposition) in the postoperative CT images rendered the evaluation too inaccurate. Finally, four cut planes (one cut plane of patients numbers 4, 5, 7, and 10) were excluded because the presence of the metallic prosthesis rendered the postoperative CT images unsuitable for the evaluation of these bone cuts.

The errors in safe margin (difference between achieved and desired resection margins) averaged −0.8 mm (95% CI: −1.8 to 0.1 mm). The maximum positive error (cutting over the desired resection margin) was 0.3 mm (patient number 7), while the maximum negative error (cutting under the desired resection margin) was −3.4 mm (patient number 5).

The location accuracy of the achieved cut planes with respect to the desired target planes averaged 2.5 mm (95% CI: 1.8 to 3.2 mm). The maximum inaccuracy was found for patient number 5 with a difference of 4.4 mm between desired and achieved cut planes.

## 4. Discussion

This study reported a clinical series of 11 PSI-assisted bone tumor resections within the pelvis. The observed results showed that PSI-assisted bone-cutting can be performed safely with an accuracy clinically relevant for bone tumor surgery within the pelvis.

Histopathological results of the resected tumor specimens did not reveal any marginal or intralesional resection. However, for patient number 8, the resected tumor specimen had to be suddenly extracted because of severe intraoperative bleeding but could not have been removed* en bloc* because of its complex 3D shape, and the surgeon had to urgently morselize the specimen to be able to extract the tumor as soon as possible, inevitably inducing R2 bone resection margin. Adequate orientation of the desired resection planes should be optimized preoperatively during the planning process to guarantee intraoperative rapid* en bloc* extraction of the tumor. Moreover, for patient number 5, the iliac bone resection margin was classified R0 but the patient had a soft tissue local recurrence in the external iliac vessels that has been classified R1. In such case, achieving adequate soft tissue margin is highly challenging, especially as PSI is a technology that assists bone-cutting with no intended action on soft tissues.

By systematically achieving clear bone margins, it appears that PSI technology could have the potential to significantly reduce the risk of local recurrence. However, the short-term follow-up of the present study is not sufficient to state any improvement in terms of the oncological outcomes. A minimum 3-year follow-up should enable the drawing of more stringent conclusions about the presence or absence of local recurrence after bone tumor resections [44]. Moreover, local recurrence can appear even if safe margins are achieved, as it was recently demonstrated in several case series of navigation-assisted bone tumor surgeries [18, 44, 45].

Results in terms of the errors in safe margin ESM or the location accuracy L demonstrated how PSI enabled the surgeon to intraoperatively replicate the resection strategies with a very good cutting accuracy. These findings are consistent with the levels of bone-cutting accuracy already published in the literature on the clinical use of PSI and navigation technologies for bone tumor surgery. Wong et al. [35] reported a millimetric accuracy during a PSI-assisted bone tumor resection of the femur. Ritacco et al. [20] reported a series of 28 navigation-assisted bone tumor resections with an average cutting error of 2.5 mm between planned and achieved resection planes. Finally, Khan et al. [36] also investigated bone-cutting accuracy in accordance with the ISO1101 standard and reported a 2 mm location accuracy during a PSI-assisted multiplanar resection on a cadaveric femur.

Improvements in accuracy observed here are consistent with findings of a previous study on synthetic pelvic bone models [37]. The observed level of accuracy suggests that a 5 mm safe margin should be sufficient to obtain clear surgical margins when using PSI since the observed maximum error in safe margins is 3.4 mm. This decrease in the level of desired safe margins allows performing resections closer to the tumor boundary, offering the possibility of preserving either the sacroiliac or hip joint, a portion of bone, muscle insertions, or nerve roots. For example, in patient number 11, the 5 mm margin enabled preserving three sacral nerves, keeping organ functionalities of the patient.

PSI have several potential advantages that are more difficult to assess objectively. First, PSI are cost-effective since the technique is pay-per-use and does not require any intraoperative assistance. Second, in addition to the improvements in bone-cutting accuracy, the direct visual control of the cutting depth provided by the PSI through calibrated rulers allows for an easy mobilization of the resected tumor specimen, potentially increasing the safety of critical bone cuts such as posterior transsacral bone cuts.

PSI technology has some limitations. It requires a multidisciplinary team and, particularly, a technical person to perform the preoperative planning and design the instruments. PSI requires bone exposure to find a stable bone surface and to be accurately positioned. This can be a limit to the technique but somehow moderate since the bone exposure is also required before cutting bones with the conventional technique. Bone exposure was limited thanks to the visual support of the 3D bone model that was provided with the PSI. Finally, mispositioning of PSI can occur leading to an inaccuracy during the bone-cuttings.

PSI have to respect some technical requirements to meet relevant surgical performances. First, PSI must optimally fit into the bone surface without interfering with soft tissues (ligaments, muscles insertions, etc.). Then, the stability of PSI should be sufficient to ensure a safe and quick positioning on the bone surface. An interesting method to determine the stability of the PSI has been recently proposed in [46]. These authors have developed a quantitative stability score that provides an objective evaluation of the stability according to the contact surfaces between PSI and bone. This score can be computed before manufacturing to ensure an adequate stability. Finally, preoperative workflow requires sending images, defining surgical strategy, planning resection, and finally designing and manufacturing PSI. The timeframe for planning and manufacturing PSI is about 4 weeks, which fits with the clinical situation when patients undergo neochemotherapy and/or radiotherapy. When chondrosarcomas do not require neither chemotherapy nor radiotherapy, the complete process can be significantly shortened, requiring a high responsiveness of both surgeon and engineer. Anyway, a strong collaboration between surgeon and engineer as well as efficient communication tools are required.

This study has some limitations. First, this study has no randomization or control group. The rarity of bone tumors does not allow us to perform such a randomized study. Second, the follow-up period of this study is short so that no stringent conclusion about survival and local recurrence rates could be drawn reasonably. Third, the accuracy evaluation process proposed in this study is prone to some types of methodological errors that are hardly controllable. For example, the postoperative CT images can be unsuitable for evaluation purposes because the presence of a metallic implant (used for reconstruction) renders the identification of the cutting planes too inaccurate. Also, the 3D model reconstructed from the postoperative CT images has to be registered with the preoperative CT images and may lead to registration errors. Finally, bone formation may occur between the time of the surgery and the time of the postoperative CT acquisition, thus altering the identification of the achieved cut planes and potentially overestimating the cutting errors.

## 5. Conclusion

The present clinical study demonstrated that using PSI during bone tumor resection within the pelvis provides good cutting accuracy. Intraoperative use of PSI appeared to be quick and easy-to-handle and allowed obtaining bone clear margins. Follow-up should continue to observe local recurrence rate and draw stronger conclusion about the use of PSI technology during bone tumor resection within the pelvis and its effect on clinical outcomes.

## Figures and Tables

**Table 1:** Patient series, tumor data, histopathological resection results and clinical outcomes.

Patient (gender (M/F), age (years))	Histology^1^	Enneking zones	Tumor size (mm)	Number of resection planes^2^	Closest desired safe margins^3^ (mm)	Histological analysis	Neo and adjuvant treatment	Reconstruction	Complication	Follow-up (months)	Current status^4^
1 (F, 76)	CHS grade 2	I + II	200	1 (HER)	10	R0	—	Hip transposition	Deep infection	28	DF
2 (M, 54)	CHS grade 2	I + II + III	120	1 (HER)	6	R0	—	Prosthesis	—	19	DF
3 (M, 57)	CHS grade 2	II	140	3	10	R0	—	Prosthesis	—	17	DF
4 (M, 65)	CHS grade 2	II + III	160	2 (HER)	10	R0	—	Prosthesis	Deep infection	16	DF
5 (F, 69)	LMS	I + II	150	2	10	R0∗	Chemotherapy	Prosthesis	ST LR∗∗∗	22	DF
6 (M, 66)	CHS grade 2	II	140	3 (HER)	—	R0	—	Prosthesis	Deep infection	10	DF
7 (M, 60)	CHS grade 2	I + II + III	170	2	5	R0	—	Prosthesis	Deep infection; hip dislocation	12	DF
8 (F, 27)	CHS grade 2	IV	60	4	7	R2∗∗	—	—	Scare desunion	8	DF
9 (M, 46)	CHS grade 2 LR of myxoid	I + II	270	1	3	R0	—	Prosthesis	Deep infection; hip dislocation	7	DF
10 (M, 17)	ES	II + III	100	3	10	R0	Chemotherapy; radiotherapy	Prosthesis	—	4	DF
11 (M, 54)	Bone sarcoma	IV	100	4	5	R0	Chemotherapy	—	—	0	DF

^1^CHS = chondrosarcoma; LMS = leiomyosarcoma; ES = Ewing's sarcoma; LR = local recurrence.

^2^HER = hip extra-articular resection.

^3^See Table 2 for detailed data on bone-cutting accuracy.

^4^DF = alive disease-free.

∗R0 bone resection margin but R1 soft-tissue resection margin.

∗∗R2 bone resection margin because tumor has been morselized for extraction.

∗∗∗Soft-tissue local recurrence at 18 months; patient was reoperated on; now patient is free of disease.

**Table 2:** Achieved surgical margins SM and location accuracy L.

Resection plane (patient)	Desired safe margin (mm)	Achieved surgical margin SM (mm)	Error in safe margin ESM (mm)	Location accuracy L (mm)
1 (2)	6	5.2	−0.8	2.1
2 (4)	15	14.2	−0.8	2.5
3 (5)	10	6.6	−3.4	4.4
4 (7)	10	10.3	0.3	1.1
5 (9)	3	2.8	−0.2	2.8
6 (10)	12	12.1	0.1	2.7
7 (10)	10	8	−2	1.5
8 (11)	5	3.5	−1.5	2.7
9 (11)	5	5.7	0.7	2.6
